# DCLK1 Regulates Pluripotency and Angiogenic Factors *via* microRNA-Dependent Mechanisms in Pancreatic Cancer

**DOI:** 10.1371/journal.pone.0073940

**Published:** 2013-09-09

**Authors:** Sripathi M. Sureban, Randal May, Dongfeng Qu, Nathaniel Weygant, Parthasarathy Chandrakesan, Naushad Ali, Stan A. Lightfoot, Panayotis Pantazis, Chinthalapally V. Rao, Russell G. Postier, Courtney W. Houchen

**Affiliations:** 1 Department of Medicine, the University of Oklahoma Health Sciences Center, Oklahoma City, Oklahoma, United States of America; 2 Department of Pathology, the University of Oklahoma Health Sciences Center, Oklahoma City, Oklahoma, United States of America; 3 Department of Surgery, the University of Oklahoma Health Sciences Center, Oklahoma City, Oklahoma, United States of America; 4 Department of Veterans Affairs Medical Center, Oklahoma City, Oklahoma, United States of America; 5 The Peggy and Charles Stephenson Cancer Center, Oklahoma City, Oklahoma, United States of America; 6 COARE Biotechnology Inc., Oklahoma City, Oklahoma, United States of America; University of Kentucky, United States of America

## Abstract

Stem cell pluripotency, angiogenesis and epithelial-mesenchymal transition (EMT) have been shown to be significantly upregulated in pancreatic ductal adenocarcinoma (PDAC) and many other aggressive cancers. The dysregulation of these processes is believed to play key roles in tumor initiation, progression, and metastasis, and is contributory to PDAC being the fourth leading cause of cancer-related deaths in the US. The tumor suppressor miRNA *miR-145* downregulates critical pluripotency factors and oncogenes and results in repressed metastatic potential in PDAC. Additionally, the *miR-200* family regulates several angiogenic factors which have been linked to metastasis in many solid tumors. We have previously demonstrated that downregulation of DCLK1 can upregulate critical miRNAs in both *in vitro* and *in vivo* cancer models and results in downregulation of c-MYC, KRAS, NOTCH1 and EMT-related transcription factors. A recent report has also shown that Dclk1 can distinguish between normal and tumor stem cells in *Apc* ^*min/+*^ mice and that ablation of Dclk1^+^ cells resulted in regression of intestinal polyps without affecting homeostasis. Here we demonstrate that the knockdown of DCLK1 using poly(lactide-co-glycolide)-encapsulated-DCLK1-siRNA results in AsPC1 tumor growth arrest. Examination of xenograft tumors revealed, (a) increased *miR-145* which results in decreased pluripotency maintenance factors OCT4, SOX2, NANOG, KLF4 as well as KRAS and RREB1; (b) increased let-*7a* which results in decreased pluripotency factor LIN28B; and (c) increased *miR-200* which results in decreased VEGFR1, VEGFR2 and EMT-related transcription factors ZEB1, ZEB2, SNAIL and SLUG. Specificity of DCLK1 post-transcriptional regulation of the downstream targets of *miR-145*, *miR-200* and let-*7a* was accomplished utilizing a luciferase-based reporter assay. We conclude that DCLK1 plays a significant master regulatory role in pancreatic tumorigenesis through the regulation of multiple tumor suppressor miRNAs and their downstream pro-tumorigenic pathways. This novel concept of targeting DCLK1 alone has several advantages over targeting single pathway or miRNA-based therapies for PDAC.

## Introduction

Pancreatic ductal adenocarcinoma (PDAC) is the fourth leading cause of cancer-related deaths in the U.S. annually with a 5% 5-year survival rate. Despite more than 10 years of FDA-approved therapeutic regimens and marked improvements in medical and surgical care, no significant impact on PDAC patient survival has been observed [1]. Recently, a subset of cells with cancer stem-cell (CSC) properties has been identified in PDAC [2] that are capable of unlimited self-renewal and give rise to more-differentiated and more-aggressive progeny, which are often resistant to conventional chemotherapy and radiotherapy [2,3]. The inability to eradicate these CSCs is postulated to be a reason for tumor relapse, metastasis and death following initial responses to treatment [4]. Another critical obstacle in combating solid tumor cancers in general is the heterogeneity of cell types within the tumor microenvironment [5] and the highly desmoplastic tumor niche [6]. This heterogeneity is further complicated by epithelial to mesenchymal transition (EMT), a process that plays a key role in cancer invasion and metastasis [7,8]. Many of the EMT-inducing transcription factors such as SNAI1 (SNAIL), SNAI2 (SLUG), ZEB1, ZEB2, TWIST1, FOXC2 and Goosecoid have been associated with tumor invasion and metastasis [9]. Recently, a number of reports have identified the microRNA (miRNA) *miR-200* family as fundamental markers and regulators of EMT [10–12]. miRNAs are small, 19–22 nucleotide long, non-coding RNAs that inhibit gene expression at the posttranscriptional level [13]. A strong link between miRNA dysregulation and human cancer has been established [14]. Consequently miRNAs have been demonstrated to act either as oncogenes (e.g., *miR-155*, *miR-17−5p* and *miR-21*) [15,16] or tumor suppressors (e.g., *miR-34*, *miR-15a*, *miR-16−1* and let-*7*) [17–20]. However, the precise regulatory features that tip the balance towards a cancer phenotype with respect to tumor suppressor versus oncogenic miRNA expression are poorly understood.

Pluripotency is the ability of a cell to differentiate into any cell type and is a unique characteristic of embryonic stem cells (ESCs). Pluripotency transcription factors such as OCT4, SOX2, NANOG and KLF4 form regulatory networks and influence a wide spectrum of downstream genes. Overexpression of these factors can dedifferentiate human and mouse somatic cells into induced pluripotent stem cells (iPSCs) [21]. The reprogramming factors OCT4, SOX2, NANOG, KLF4, c-MYC, and LIN28 have also been suggested to be oncogenes and may be implicated in the development of several cancers [21–26]. Multiple reports have shown that these transcription factors are regulated, at least in part, by miRNAs [21,27,28]. *miR-145* specifically inhibits the aforementioned pluripotency factors by binding the 3’ untranslated region (UTR) of mRNA, which leads to inhibition of ESCs, self-renewal, and induction of differentiation. Furthermore, loss of *miR-145* impairs differentiation and elevates OCT4, SOX2, and KLF4 [21]. Additionally, it has been demonstrated that the *miR-145* promoter is bound and repressed by OCT4 in ESCs. This indicates (a) the existence of a direct link between the core reprogramming factors and *miR-145*, and (b) the presence of a double-negative feedback loop involving OCT4, SOX2, KLF4, and *miR-145* [21]. In cancer, *miR-145* is downregulated and has been demonstrated to possess tumor suppressor properties. The loss of *miR-145* (*miR-143/145* cluster) is observed in KRAS mutated pancreatic cancers, and therapeutic restoration of these miRNAs abrogates tumorigenesis [29,30]. Furthermore, Ras-responsive element binding protein 1 (RREB1) represses *miR-143*/*miR-145* promoter activity, which indicates that repression is an early event in pancreatic cancer initiation and progression [29]. Additionally, KRAS and RREB1 are targets of *miR-143/145*, demonstrating a feed-forward mechanism that potentiates RAS signaling-mediated PDAC tumor progression [29]. It has been recently demonstrated that ectopic expression of *miR-143/145* results in repressed metastasis and increased adhesion of pancreatic cancer cells [30]. These data taken together suggest that *miR-145* is a master regulator of iPSCs factors in ESCs and CSCs and may play an important role in inhibition of pancreatic cancer initiation, progression and EMT.

Vascular endothelial growth factor (VEGF) signaling plays an important role in tumor angiogenesis. VEGF family members mediate the critical signaling by binding to two tyrosine kinase receptors, VEGFR1 and VEGFR2. VEGFR1 is required for endothelial cell survival; whereas, VEGFR2 is required for receptor-mediated angiogenic and vascular permeability activity [31–33]. Recently it has been demonstrated that these angiogenic factors (VEGFR1 and 2) are involved in tumor metastasis of renal and colon carcinoma cells [34]. VEGF signaling is known to promote tumor vasculature and endothelial cell proliferation in PDAC. Studies using soluble VEGFR1 and VEGFR2 (inhibition of VEGFR-mediated signaling in a dominant-negative manner) or the VEGFR tyrosine kinase inhibitors resulted in inhibition of tumor angiogenesis, growth and metastasis in PDAC mouse models [35–38].

DCLK1 (formerly known as DCAMKL-1) is a putative intestinal and pancreatic stem cell marker and is upregulated in the stroma and epithelium of PDAC. It has also recently been described as a marker of the relatively undefined tuft/brush cell in the pancreas and intestine [39,40]. It negatively regulates several tumor suppressor miRNAs and likely plays a key role in initiation and progression of solid tumor cancers [11,41]. In addition, it regulates several key oncogenes (c-MYC, KRAS and NOTCH1) and EMT. Moreover, overexpression of pluripotency factors in liver cancer cells resulted in increased expression of DCLK1 [42]. Recently Nakanishi et al. [43], have used an elegant Dclk1-Cre mouse model to demonstrate, that Dclk1 marks intestinal tumor stem cells. Ablation of Dclk1 expressing cells in *Apc* ^*min/+*^ mice resulted in regression of polyps without any damage to the normal intestinal epithelium. In this report, following the knockdown of DCLK1 using nanoparticle-encapsulated siRNA (NPsiDCLK1) in tumor xenografted mice, we observed a significant increase in: (A) *miR-143/145* cluster, which resulted in downregulation of key pluripotency markers (OCT4, SOX2, KLF4 and NANOG); (B) let-*7a*, which resulted in decreased pluripotency factor LIN28B; and (C) *miR-200a, b* and *c*, which resulted in downregulation of EMT and angiogenic factors. Administration of NPsiDCLK1 did not result in overt toxicity in mice. These data taken together suggest a central regulatory role of DCLK1 in pancreatic tumorigenesis.

## Materials and Methods

### Small interfering RNAs

DCLK1 siRNA (siDCLK1) sequence targeting the coding region of DCLK1 (accession No. NM_004734) (GGGAGUGAGAACAAUCUACtt) and scrambled siRNAs (si-SCR) not matching any of the human genes were obtained (Ambion Inc, Austin, TX).

### Synthesis and characterization of DCLK1 siRNA NPs

Poly(lactide-co-glycolide) acid nanoparticles (PLGA NPs) were synthesized using a double emulsion solvent evaporation technique as described earlier [11,44]. Briefly, siRNA (DCLK1 or scrambled) was condensed on the cationic polymer poly(ethyleneimine) (PEI) to form an siRNA-PEI complex. This complex was added to PLGA in chloroform and vortexed and transferred to 2% polyvinyl alcohol. This emulsion was sonicated and allowed to evaporate overnight. The size, polydispersity index, and zeta-potential measurements of synthesized siRNA NPs were determined using diffraction light scattering (DLS) utilizing Zeta PALS (Brookhaven Instruments, Holtsville, NY).

### Xenograft tumor model

NOD/SCID mice were purchased from the Jackson Laboratory (Bar Harbor, Maine) and housed in pathogen-free conditions. They were cared for in accordance with guidelines set forth by the American Association for Accreditation of Laboratory Animal Care and the U.S. Public Health Service Commissioned Corps’ “Policy on Human Care and Use of Laboratory Animals." All studies were approved and supervised by the University of Oklahoma Health Sciences Center’s Institutional Animal Care and Use Committee (IACUC). AsPC-1 cells (1 × 10^7^) were injected subcutaneously into the flanks of 4- to 6-wk-old mice (n=3). Tumors were measured using a caliper and the volume was calculated as (length × width^2^) × 0.5. The tumors were palpable 30 days after injection of cells. NPs were reconstituted in sterile normal saline and injected directly into the tumors. Each animal bearing the tumor was injected on days 30, 33, 36, 39, and 42 with one of the following preparations -50 µl (5 µM) of siRNA-NP preparation [NP alone (Control), NP-siScrambled (NPsiSCR), or NPsiDCLK1]. All mice were killed on day 45.

### Immunohistochemistry, Western blot, real-time reverse Transcription-PCR, miRNA analysis, Invasion assay and luciferase reporter gene assay

These analyses were carried out as previously described [11,41]. Detailed descriptions are provided in the Supplementary section of Materials and Methods (Text S1).

## Results

### RNA silencing of DCLK1 inhibits pancreatic cancer xenograft growth

**Figure 1:**
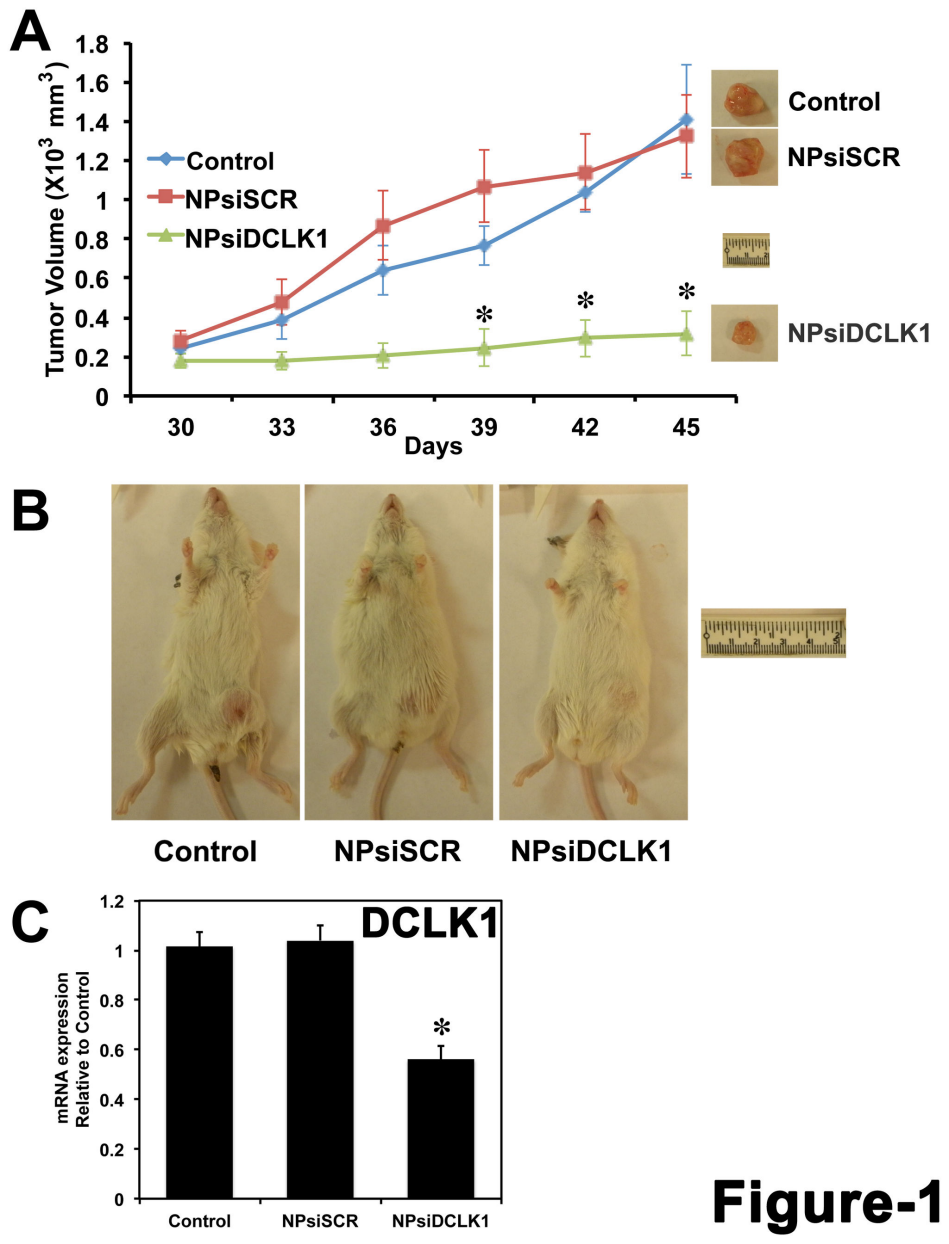
DCLK1 is essential for human pancreatic tumor xenograft growth. A, AsPC-1 human pancreatic cancer cells were subcutaneously injected into the flanks of NOD/SCID mice to generate tumors. At day 30, PLGA NP encapsulated siRNAs (siDCLK1 and siSCR) were injected directly into the tumors and followed by injections every third day. After 5 injections, tumors were excised at day 45 and are represented above. Tumor volume was measured every 3 days. B, Representative photograph of mice bearing the tumors from each group are shown. C, The expression of DCLK1 mRNA in the tumors quantitated by real-time RT-PCR. Values are given as average ± SEM, and *asterisks* denote statistically significant differences (*p < 0.01) compared with Control (NP alone).

AsPC-1 human pancreatic cancer cells were injected subcutaneously into the flanks of NOD/SCID mice and tumors were allowed to develop for 30 days. NP encapsulated siRNAs (NPsiDCLK1 and NPsiSCR) were injected intratumorally. Nanoparticle encapsulation was performed to overcome the theoretical limitations of siRNA-based delivery [45]. The efficacy of this approach has been previously tested in colorectal cancer xenografts [11]. On day 30, when the tumors were palpable, the treatments were initiated and continued every third day for a total of five injections. Tumors were excised at day 45, and the tumor volumes are represented in Figure 1. Administration of NPsiDCLK1 resulted in a significant (~85%) reduction (*p* < 0.01) in tumor volume compared with either the Control (NPs-alone) or NPsiSCR-treated tumors (Figure 1A and 1B). mRNA analysis demonstrated a significant downregulation (*p* < 0.01) of DCLK1 mRNA compared to Control or NPsiSCR treated tumors (Figure 1C). These data taken together demonstrate that inhibition of DCLK1 results in AsPC-1 tumor xenograft growth arrest.

### Knockdown of DCLK1 results in downregulation of pluripotency factors in pancreatic tumor xenografts via miR-145

**Figure 2:**
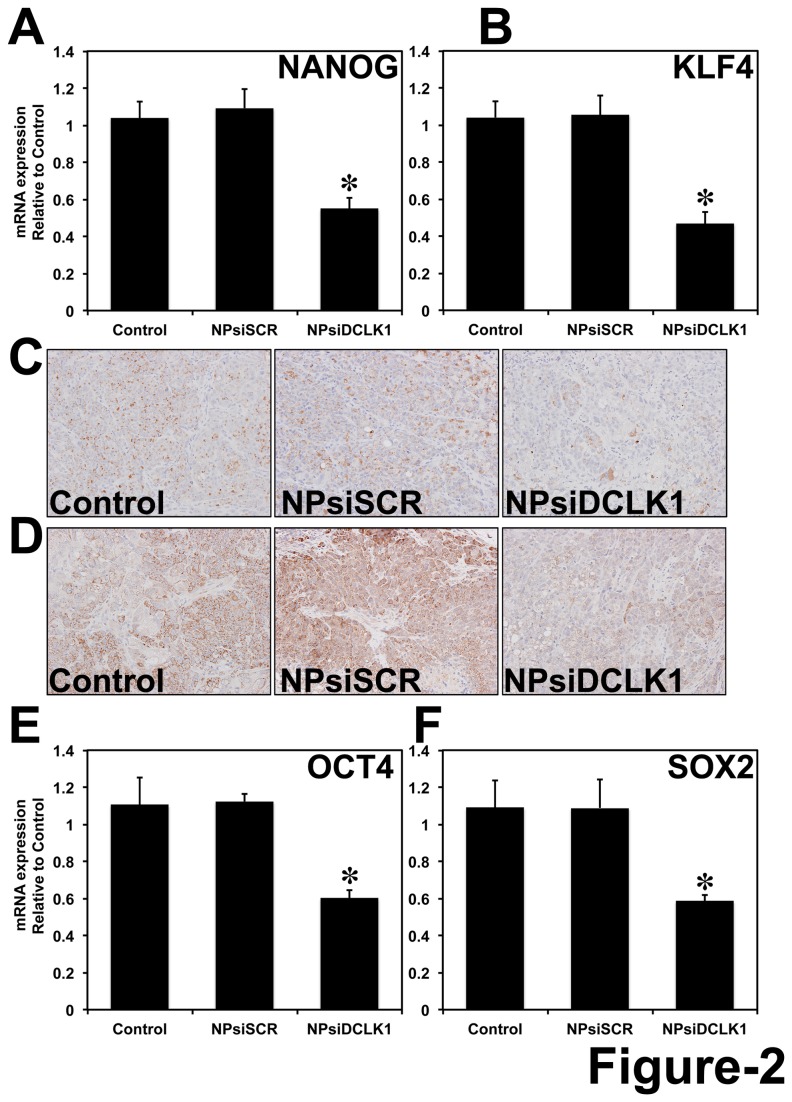
DCLK1 regulates pluripotency. siRNA-mediated knockdown of DCLK1 resulted in downregulation of pluripotency factors: NANOG mRNA (A); KLF4 mRNA (B); NANOG protein (C); KLF4 protein (D); OCT4 mRNA (E) and SOX-2 mRNA (F). mRNA was analyzed using real-time RT-PCR and protein by immunohistochemical analyses. For bar graph in A, B, E and F, values are given as average ± SEM, and *asterisks* denote statistically significant differences (*p < 0.01) compared with Control (NP alone).

It has been demonstrated that OCT4, SOX2, c-MYC, LIN28, NANOG and KLF4 are required for ESC self-renewal and pluripotency and are upregulated in some aggressive cancers and in CSCs [23–26,28]. Overexpression of these factors can reprogram or dedifferentiate human and mouse somatic cells into iPSCs. Here we observed a significant (*p* < 0.01) downregulation (>40%) in the mRNA expression of pluripotency markers NANOG and KLF4 (Figure 2A and 2B) by real-time RT-PCR following the knockdown of DCLK1. Similarly NANOG and KLF4 proteins were also downregulated as analyzed by immunohistochemical analysis (Figure 2C and 2D). Additionally, we also observed a significant (>40%) downregulation of OCT4 (Figure 2E) and SOX2 (Figure 2F) at the mRNA level following the knockdown of DCLK1 in AsPC-1 tumor xenografts.

### DCLK1 post-transcriptionally regulates miR-145 in pancreatic cancer

**Figure 3:**
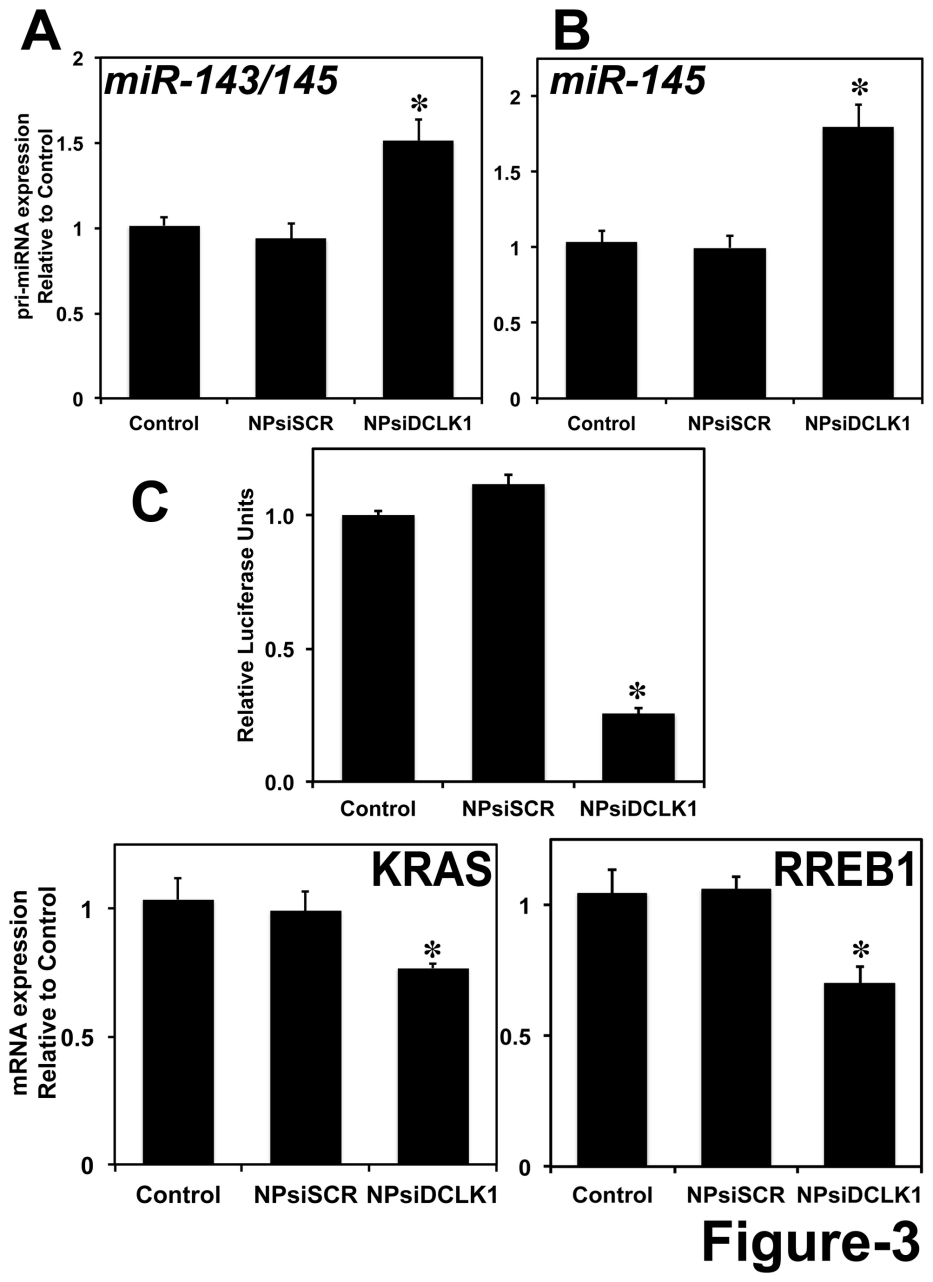
DCLK1 negatively regulates *miR-143/145*. Following the knockdown of DCLK1 in AsPC-1 tumor xenografts, a significant upregulation of *miR-143/145* cluster (A) and *miR-145* miRNA (B) by real-time RT-PCR. C, A decrease in luciferase activity (luciferase units) following transfection with plasmid-encoding luciferase containing the *miR-145* binding site was observed following the knockdown of DCLK1 in AsPC-1 human pancreatic cancer cells. Knockdown of DCLK1 also resulted in the downregulation of KRAS (D) and RREB1 (E) mRNA, downstream targets of *miR-143/145* miRNA cluster, analyzed using real-time RT-PCR. Values are given as average ± SEM, and *asterisks* denote statistically significant differences (*p < 0.01) compared with Control (NP alone).

*miR-145* has been shown to repress OCT4, SOX2, and KLF4, thereby repressing pluripotency and controlling differentiation [21]. *miR-145* is downregulated in various cancers and has been demonstrated to possess tumor suppressor and metastasis inhibitory properties [29,30]. Earlier reports [11,28,41] and the data presented above indicate that DCLK1 negatively regulates tumor suppressor miRNAs like let-*7a*, *miR-144* and *miR-200a*. Similarly, here we observed a significant induction (1.5-fold) of *pri-miR-143/145* cluster miRNA (Figure 3A) and *pri-miR-145* miRNA (Figure 3B) following the knockdown of DCLK1 in AsPC-1 tumor xenografts. Furthermore, we performed a luciferase reporter gene assay to quantitatively measure the effect of DCLK1 knockdown on *miR-145* miRNA. AsPC-1 cells were transfected with a plasmid containing the firefly luciferase gene with a complementary *miR-145* binding site at the 3' UTR. Following transfection, cells were treated with NPs alone, NPsiSCR, or NPsiDCLK1 and were subjected to luciferase activity measurement. A reduction in luciferase activity was observed in cells treated with NPsiDCLK1 compared to the control or NPsiSCR (Figure 3C). These data suggest that knockdown of DCLK1 results in downregulation of *miR-145* miRNA downstream targets in pancreatic cancer cells. It has been previously demonstrated that a feedback loop mechanism exists between *miR-143/145* and KRAS and RREB1. RREB1 is known to repress the transcription of *miR-143/145* by binding to its promoter region. Additionally, KRAS and RREB1 are downstream targets of *miR-143/145* [29]. Knockdown of DCLK1 resulted in increased expression of *miR-143/145* cluster and downregulation of its downstream target KRAS (Figure 3D). Furthermore, we found that the expression of RREB1 was significantly downregulated following the administration of siRNA against DCLK1 (Figure 3E). With these data, we speculate that DCLK1 plays a role in post-transcriptional regulation of *miR-143/145* cluster and thereby downregulates KRAS and RREB1 in pancreatic tumor xenografts.

Overall, these data taken together demonstrate a potential regulatory role for DCLK1 in the expression of iPSC factors in pancreatic cancers via *miR-145* miRNA.

### DCLK1 regulates let-**7a** and its downstream target LIN28B in pancreatic cancer

**Figure 4:**
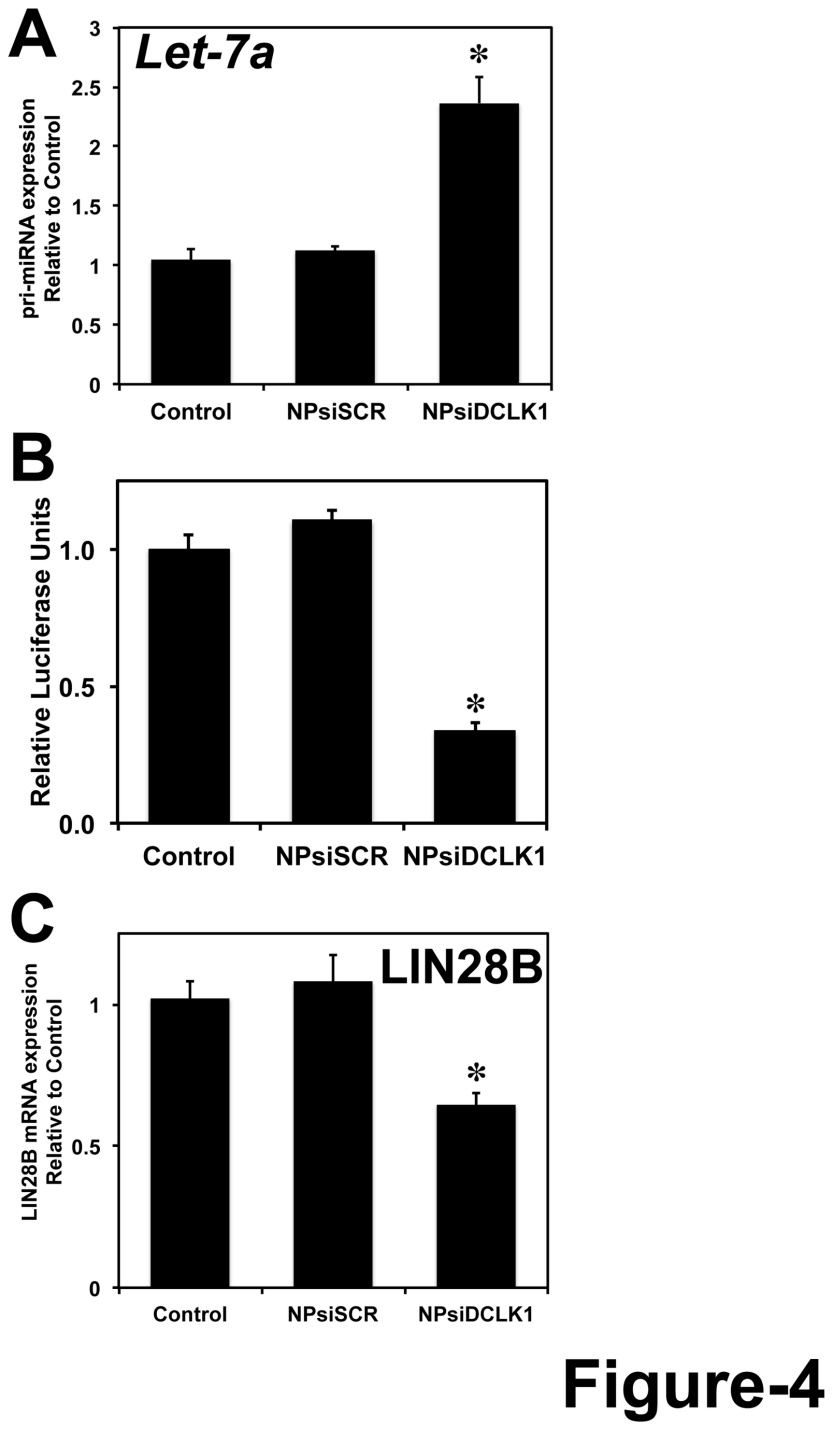
DCLK1 regulates LIN28B via let-*7a*. A, siRNA-mediated knockdown of DCLK1 in tumor xenografts results in increased expression of *pri-let-7a* miRNA. B, Following the knockdown of DCLK1, a decrease in *miR-let7a* dependent luciferase activity was observed in AsPC-1 cells. C, LIN28B mRNA downstream target of let-*7a* was downregulated in tumors treated with NPsiDCLK1. Values in the bar graphs are given as average ± SEM, and asterisks denote statistically significant differences (*P < 0.01) compared with Control (NP alone).

In pancreatic and colorectal cancer cells, we have previously demonstrated that DCLK1 negatively regulates miRNA let-*7a*. siRNA-mediated knockdown of DCLK1 resulted in downregulation of let-*7a* downstream targets c-MYC and KRAS. In pancreatic tumor xenografts treated with NPsiDCLK1, we observed a significant upregulation of let-*7a* (Figure 4A) and subsequent downregulation of its downstream target c-MYC (mRNA and protein) (Figure S1). Reports suggests that LIN28B, a pluripotency maintenance factor regulates miRNA let-*7* and in turn let-*7* regulates LIN28B (due to the presence of binding site in the 3’ UTR of LIN28B), which suggests a double negative feedback loop between LIN28 and let-*7* [46,47]. Here we wanted to see whether NPsiDCLK1 regulates downstream targets of let-*7a* miRNA. AsPC-1 cells were transfected with plasmid encoding firefly luciferase gene under the control of 3’ UTR containing binding site for let-*7*. Following the transfection, the cells were treated with NPsiDCLK1 and subjected to luciferase measurement. Following the knockdown of DCLK1, we observed a significant downregulation of *let-7*-dependent luciferase activity (Figure 4B) indicating that NPsiDCLK1 regulates downstream targets of let-*7* in pancreatic cancer cells. Based on real-time RT-PCR analysis of tumors treated with NPsiDCLK1, we observed a significant downregulation of LIN28B mRNA compared to NPsiSCR or control-treated tumors (Figure 4C). These data indicate that DCLK1 regulates LIN28B via *let-7*-dependent mechanism.

### DCLK1 regulates miR-200 family genes and EMT in pancreatic cancer

**Figure 5:**
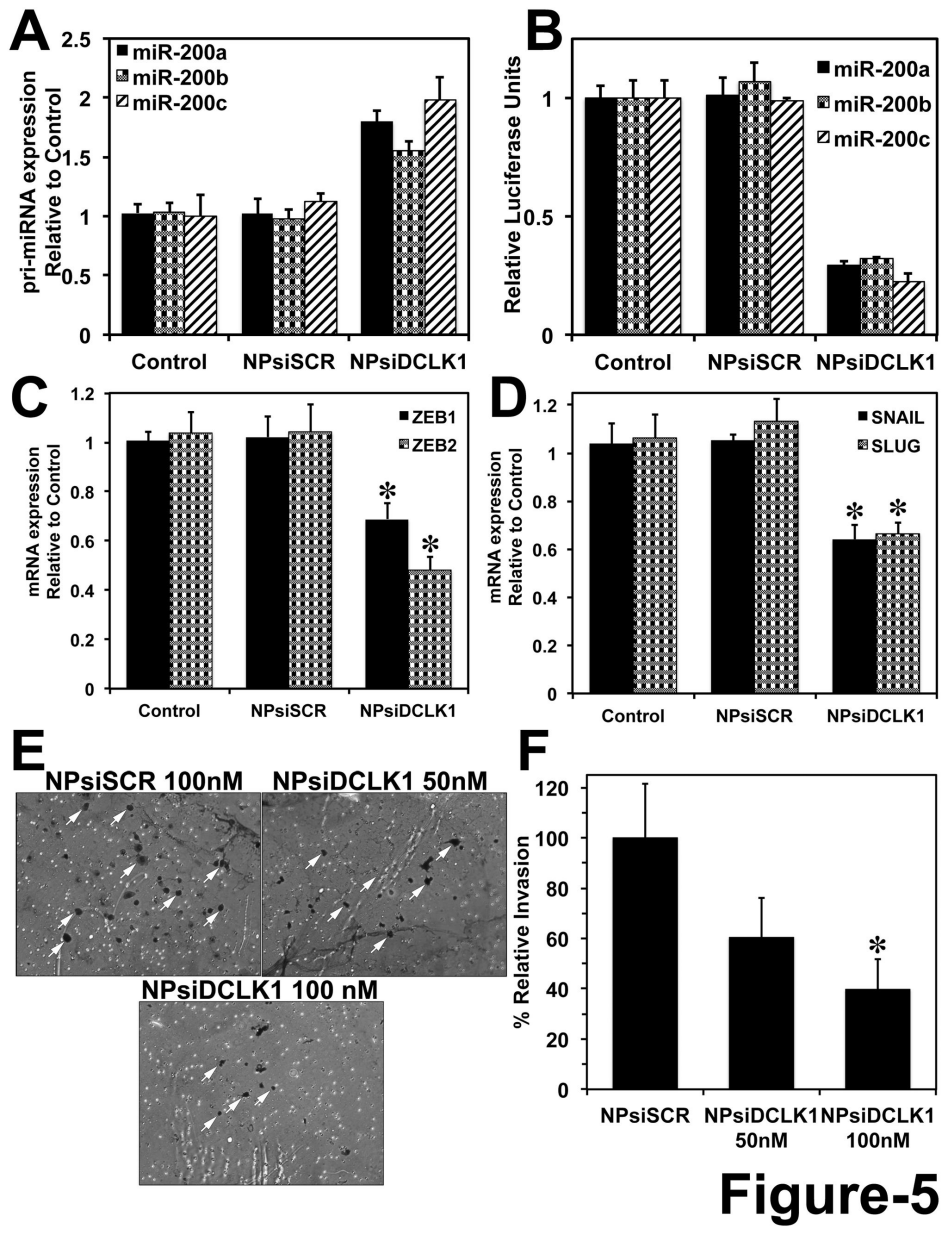
DCLK1 negatively regulates *miR-200* and inhibits EMT and invasion. A, siRNA-mediated knockdown of DCLK1 results in increased expression of *pri-miR-200a*, *pri-miR-200b* and *pri-miR-200c* by real-time RT-PCR. B, Following the knockdown of DCLK1, a decrease in *miR-200a*, *miR-200b* and *miR-200c* dependent luciferase activity was observed in AsPC-1 cells. Tumor xenografts treated with NPsiDCLK1 demonstrated a downregulation of EMT transcription factors ZEB1 and ZEB2 mRNA (C), decreased SNAIL and SLUG mRNA expression (D). E, siRNA-mediated inhibition of DCLK1 results in inhibition of invasion in AsPC-1 cells. White arrows indicate the cells invaded through the Matrigel matrix. F, Bar graph represents quantification of invaded cells following each treatment. Cells were counted in 5 different fields for each insert. Values in the bar graphs are given as average ± SEM, and asterisks denote statistically significant differences (*P < 0.01) compared with Control (NP alone).

EMT is a highly conserved process, characterized by the phenotypic conversion of epithelial cells to mesenchymal cells [7]. EMT is essential in various process including organ morphogenesis, wound healing, cancer metastasis and tissue remodeling in embryonic development. Recent studies have demonstrated that *miR-200a, b* and *c* (*miR-200*) are known to regulate EMT and angiogenesis [48]. Previous studies have demonstrated that DCLK1 is overexpressed in human pancreatic cancer tissues and co-localizes with SNAIL and SLUG. Following the knockdown of DCLK1, a significant downregulation of ZEB1, ZEB2, SNAIL and SLUG was observed following increased expression of *pri-miR-200a* in AsPC-1 cancer cells [11]. Similarly in AsPC-1 tumor xenografts, a 2-fold induction of *pri-miR*-200*a* (*p* < 0.01) (Figure 5A) was observed. Furthermore, we wanted to investigate the effect of DCLK1 knockdown on expression of *miR-200b* and *c* in AsPC-1 tumor xenografts. Similar to *miR-200a*, we observed a significant upregulation of *miR-200b* (1.5-fold) (Figure 5A) and *miR-200c* (2-fold) (Figure 5A) following the knockdown of DCLK1. Next, we wanted to investigate whether DCLK1 regulates the downstream targets of *miR-200*. We performed a luciferase reporter assay for *miR-200*. AsPC-1 cells were transfected separately with plasmids encoding the luciferase gene under the regulation of miRNA binding sites (three plasmids each containing binding sites for *miR-200a, b* or *c*) at its 3’ UTR. Following transfection, we treated the cells with NP-siDCLK1. The cell lysates were subjected to luciferase measurement. Following the knockdown of DCLK1, there was a significant downregulation of *miR-200a*, *miR-200b* and *miR-200c* (Figure 5B) mediated luciferase activity. These data indicate that DCLK1 regulates *miR-200* and its downstream targets in PDAC. We also observed a subsequent reduction of *miR-200* downstream targets ZEB1 and ZEB2 (Figure 5C), SNAIL and SLUG (Figure 5D) following the knockdown of DCLK1 in pancreatic tumor xenografts. Furthermore AsPC-1 cells were treated with NPsiSCR or NPsiDCLK1 and subjected to invasion assay using BD Biosciences Matrigel invasion chambers (BD BioCoat^TM^). We observed significant inhibition of invasion following the knockdown of DCLK1 (Figure 5E and F). These data taken together indicate that knockdown of DCLK1 inhibits EMT and invasion by regulating *miR-200* in human pancreatic tumor xenografts and cancer cells. Similarly to our previous studies, following the knockdown of DCLK1, we also observed inhibition of NOTCH1 via *miR-144* (Figure S2) in AsPC-1 tumor xenografts.

### DCLK1 regulates angiogenic factors in PDAC

**Figure 6:**
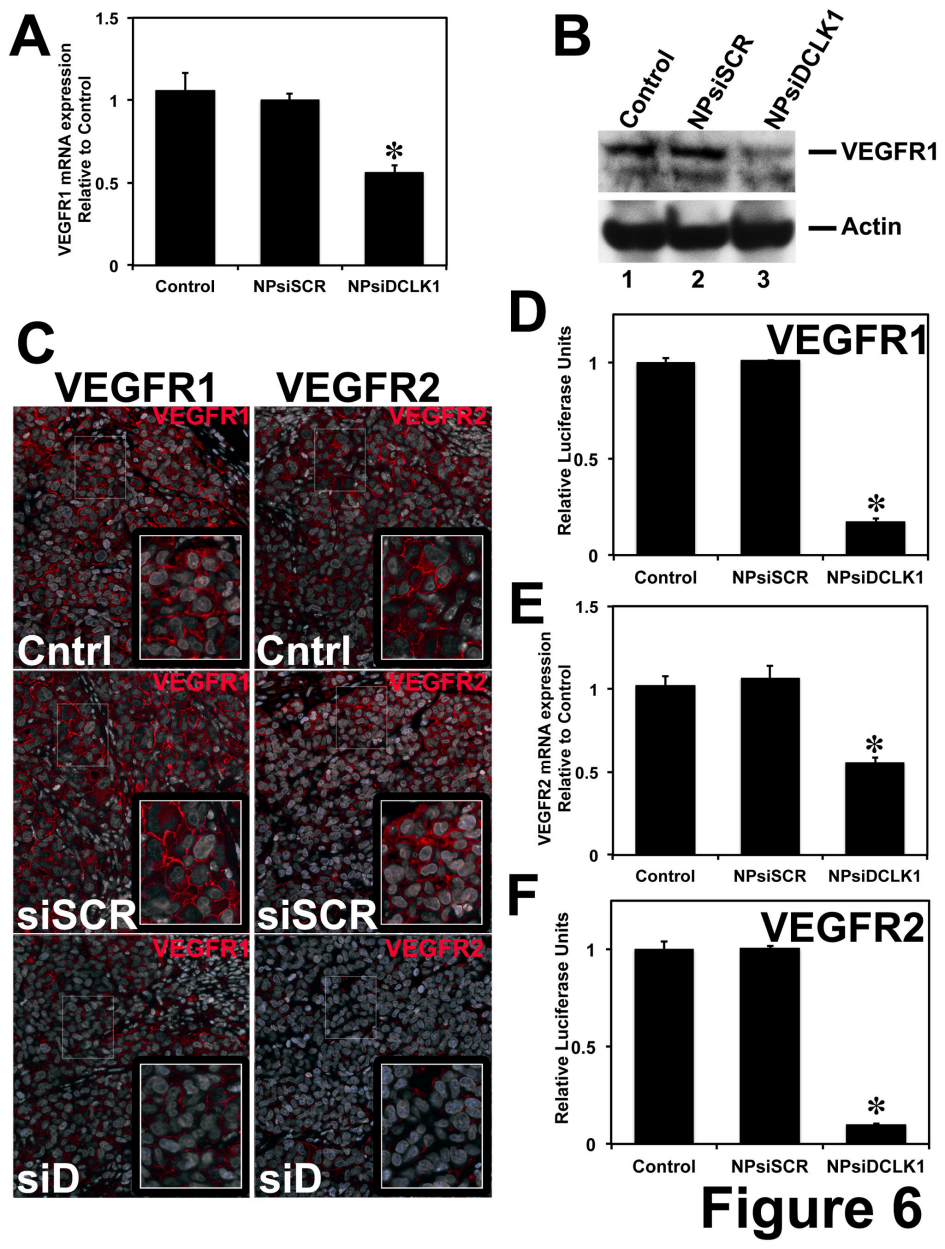
DCLK1 regulates angiogenic factors. A, siRNA-mediated knockdown of DCLK1 results in decreased expression of VEGFR1 mRNA in NPsiDCLK1 treated tumor xenografts. A decrease in VEGFR1 protein was observed in NPsiDCLK1 treated tumors (B – Western blot; C – Immunofluorescence – left panel, VEGFR1 red). D, Following the knockdown of DCLK1, a decrease in VEGFR1-3’UTR-dependent luciferase activity was observed in AsPC-1 cells. E, Knockdown of DCLK1 results in decreased expression of VEGFR2 mRNA in NPsiDCLK1 treated tumor xenografts. F, A decrease in VEGFR2-3’UTR-dependent luciferase activity was observed in AsPC-1 cells. NPsiDCLK1-mediated knockdown of DCLK1 resulted in decreased expression of VEGFR2 protein estimated using immunofluorescent analysis (C – right panel, VEGFR2 red). Values in the bar graphs in A, B, D and E, are given as average ± SEM, and asterisks denote statistically significant differences (*P < 0.01) compared with Control (NP alone).

It has been demonstrated that *miR-200* inhibits lung adenocarcinoma invasion and metastasis by targeting VEGFR1. A putative binding site for *miR-200* was observed in the 3’ UTR of VEGFR1, and it was demonstrated that *miR-200* negatively regulates VEGFR1 [48]. Additionally, a recent study demonstrated that VEGFR1 and VEGFR2 has a binding site for *miR-200b* and based on luciferase-based reporter assay *miR-200b* regulates these angiogenic factors [49]. These data indicate that VEGFR1 and VEGFR2 are downstream targets of *miR-200*. Here, we found DCLK1 regulating *miR-200* and its downstream targets. We wanted to further investigate the effect of DCLK1 knockdown on angiogenic factors VEGFR1 and VEGFR2. We observed a significant downregulation of VEGFR1 mRNA (Figure 6A), protein (Figure 6B and C – left panel) in tumors treated with NPsiDCLK1 compared to control and NPsiSCR treated-tumors. We also observed downregulation of VEGFR2 mRNA (Figure 6E) and protein (Figure 6C – right panel) in pancreatic tumor xenografts treated with NPsiDCLK1. Additionally, we performed luciferase-based reporter assays for VEGFR1 and VEGFR2. AsPC-1 cells were transfected with luciferase gene with VEGFR1 or VEGFR2 3’ UTR, treated with NPsiDCLK1 and subjected to luciferase activity measurement. Following the knockdown of DCLK1, we observed a significant downregulation of VEGFR1 (Figure 6D) and VEGFR2 (Figure 6F) 3’ UTR mediated luciferase activity. These data taken together indicate that DCLK1 regulates VEGFR1 and VEGFR2 *via miR-200* in PDAC.

## Discussion

miRNAs are rapidly emerging as critical regulators of virtually all key cellular processes. Dysregulation of miRNAs are quite common in many human cancers including PDAC. In many tumors, there is either overexpression of so-called oncogenic miRNAs (e.g., *miR-155*, *miR-17−5p* and *miR-21*) [15,16] or downregulation of tumor suppressor miRNAs (e.g., *miR-34*, *miR-15a*, *miR-16−1* and let-*7*) [17–20]. The let-*7* and *miR-200* families are well-known regulators of key differentiation programs during development. Loss of let-*7* in cancer results in progression and dedifferentiation, and the *miR-200* family has been shown to be a key regulator of EMT. Furthermore, recent studies have linked let-*7* with stem cell maintenance and EMT. Thus it is quite possible that tumor progression may represent a process that results in progressive dedifferentiation (EMT) towards a cell type that has a stem cell-like phenotype. Moreover, this process appears to be tightly regulated by miRNA-dependent mechanisms [10,11,27,30]. DCLK1 regulates EMT in human pancreatic cancer cells *via* a *miR-200a* dependent mechanism [11] and is also a regulator of let-*7a* in pancreatic and colorectal cancer cells, which supports the concept that these miRNAs are relevant and novel targets in several solid tumor cancers [10,11,27,30,41]. DCLK1 is a putative marker of intestinal and pancreatic stem cells and is upregulated in several solid tumors providing additional evidence of its potential key role in the tumorigenic process. In this report, we have demonstrated that in addition to regulating several tumor suppressor miRNAs and downstream oncogenic targets, DCLK1 inhibition results in upregulation of miRNAs that negatively regulate several key pluripotency and pro-angiogenic factors. These data support the notion that the DCLK1 is a central regulator of the tumor process.

Repression of *miR-143* and *miR-145*, two co-transcribed miRNAs located on human chromosome 5q, has been reported in pancreatic cancer. Accumulating data suggest that they possess tumor suppressor activity [50,51]. Reduced *miR-143/145* expression is a common feature of many tumor types including colorectal carcinoma and PDAC [29,50,51]. Moreover, expression of these miRNAs inhibits proliferation and activates apoptosis of cancer cells *in vitro* and *in vivo* [29]. The *miR-143/145* cluster, cooperate and inhibit the expression of *KRAS2* and its downstream effector, RREB1 [29]. It has been recently demonstrated that systemic delivery of *miR-143/145* via nanovectors blocked the growth of MiaPaCa-2 derived subcutaneous xenografts compared with vehicle or mock nanovector empty plasmid controls [29,52]. The miRNA restitution was confirmed in treated xenografts by significant upregulation of the corresponding miRNA and significant decreases in specific miRNA targets (*KRAS2* and *RREB1* for *miR-143/145*). Here, we observed downregulation of c-MYC and KRAS via let-*7a* in AsPC1 tumor xenografts following the knockdown of DCLK1 (Figure S1 and Figure 3D) (a similar mechanism was previously demonstrated in pancreatic cancer cells).

Epidermal growth factor receptor (EGFR) is upregulated in various cancer including pancreatic [53]. Additionally, inhibition of EGF signaling leads to inhibition of cancer initiation and progression [54]. Recently, it was reported that *miR-145* inhibits cell proliferation of human lung adenocarcinoma by targeting EGFR, indicating that *miR-145* is a tumor suppressor miRNA [55]. Furthermore, in another published work, it was demonstrated that EGFR suppress *miR-143* and *miR-145* in a murine models of colon cancer [56]. These data taken together indicate that there is a negative feedback loop mechanism between EFGR and *miR-143/145* similar to KRAS/RREB1 and *miR-143/145*. Following the knockdown of DCLK1, we observed increase expression *miR-143/145* and significant downregulation of KRAS and RREB1. We expect EGF signaling will also be downregulated following the knockdown of DCLK1.

VEGF and its receptors VEGFR1 and VEGFR2 have been demonstrated to play an important role in tumor vascular growth, angiogenesis, and metastasis [34]. Angiogenic factors are upregulated in various cancers such as colorectal, breast, renal, liver, and ovarian and have been correlated with poor prognosis. Though PDAC is not a grossly vascular tumor, they exhibit endothelial cell proliferation, one of the mechanisms that increase angiogenesis [38]. Inhibition of VEGF-A, VEGFR1 and VEGFR2 resulted in inhibition of tumor growth and angiogenesis in mouse models of PDAC [35–38]. Based on previous studies and computational analysis of the 3’ UTR of VEGFR1 and VEGFR2, we observed a putative binding site for *miR-200* (*miR-200a, b* and *c*) [48,49]. Following the knockdown of DCLK1, *miR-200* miRNAs were upregulated and resulted in inhibition of its downstream targets (EMT-transcription and angiogenic factors). Taken together, knockdown of DCLK1 is beneficial in preventing EMT, metastasis and angiogenesis.

These exciting new data illustrate the enormous potential of miRNA-based therapy. In this report, we attempt to advance this concept a bit further. Our data suggest that by down-regulating DCLK1 expression in pancreatic cancer, we are directly inducing or facilitating upregulation of several endogenous miRNAs within the tumors. The role of DCLK1 in the regulation of pluripotency in a cancer context is novel and may present an exciting new target for anti-cancer based therapy. Using this candidate approach, we contend that endogenous upregulation of tumor suppressors may have an advantage over small molecule inhibitor-based approaches or even protein or miRNA replacement therapies. For example, a single therapeutic miRNA may regulate hundreds of critical pathways that drive tumorigenesis; whereas, the introduction of a single DCLK1 targeted agent may induce multiple therapeutic miRNAs. This mechanism may be relatively safe if recently published reports suggesting that quiescent stem cell populations are dispensable for normal homeostatic processes but are likely activated during geno/cytotoxic injury and neoplasia are correct [39,57]. Additionally, a recent study has demonstrated that ablation of Dclk1+ cells in *Apc* ^*min/+*^ mice resulted in regression of intestinal polyps without affecting the normal intestinal homeostasis [43]. These data provide the rationale for ongoing studies that investigate the role of DCLK1 in the regulation of miRNAs in cancer.

It has been demonstrated that process of dedifferentiation and EMT may result in generation of stem cell-like properties. Overexpression of pluripotency factors or stem cell maintaining factors results in dedifferentiation of fibroblasts to iPSCs. It has been recently reported that poorly differentiated breast, glioma and bladder cancer cells express a pluripotent stem cell-like signature [58]. Furthermore, induction of EMT in mammary epithelial cells generates or expresses stem cell-like phenotypes [9]. Cells isolated based on stem cell markers (CD44^high^ and C24^low^) had undergone EMT and overexpressed EMT markers like ZEB2, SNAIL and TWIST and also had the ability to form mammospheres [9,27]. These studies provide the link between the role of pluripotency factors and EMT in cancers that we explore in this report. Here we propose the hypothesis that DCLK1-mediated overexpression of these pluripotency factors in cancer cells may drive them towards a poorly differentiated phenotype, which may in turn facilitate the process of EMT [27]. Nevertheless, in this study, inhibition of DCLK1 results in inhibition of pluripotency markers and induction of *miR-200* (EMT inhibitor) and thereby drives the cancer cells towards a differentiated state with reduced invasive properties. The inhibition of invasiveness of pancreatic cancer cells was observed in the invasion assay following the knockdown of DCLK1 (Figure 5E and F).

Overexpression of NOTCH1 led to the induction of EMT phenotype by activation of mesenchymal cell markers such as ZEB1, CD44, EpCAM, and Hes-1 [59]. Moreover, alteration of the *miR-200* family has been found to be associated with the NOTCH signaling pathway in pancreatic CSCs. Furthermore, it was found that overexpression of *miR-200* family significantly inhibits the NOTCH pathway in pancreatic cancer cells, which suggests the NOTCH pathway may be one of the *miR-200* targets [60]. In this report, consistent with our previous studies, we observed downregulation of NOTCH1 in tumors treated with NPsiDCLK1 (Figure S2).

All of these studies indicate that pluripotency, angiogenesis, EMT, NOTCH1, and cancer stemness are all involved in the complex interplay of cellular signaling, which are critical features of the tumor microenvironment in pancreatic cancer that play an important role in cancer initiation, progression and metastasis. The studies presented clearly implicate DCLK1 in the regulation of *miR-143/145, miR-200*, EMT, pluripotency, angiogenesis, NOTCH1, and cancer stemness. Inhibition of multiple oncogenic pathways following the knockdown of DCLK1 was also observed in BxPC-3, human pancreatic cancer cell line (Figures S3 and S4). Finally, NPsiDCLK1 administered *via* intratumoral injection at a dose of 5 µM for 15 days had no overt toxicity in the mice, which demonstrates its safety *in vivo*. These data taken together suggest that DCLK1 plays a central regulatory role in pancreatic tumorigenesis, and the delivery of targeted siRNA based therapeutics that inhibit multiple pro-tumorigenic pathways under the control of DCLK1 may represent a simple straightforward approach to combat pancreatic cancer and perhaps other human solid tumor cancers. Finally, we contend that beyond its status as a putative marker of quiescent stem cells and/or Tuft cells, DCLK1 plays a central role in the initiation, progression and dissemination of cancer cells. This combined with its central regulatory role in stem cell-like processes, pluripotency factor regulation, EMT and angiogenesis makes it an ideal and novel target for therapy in pancreatic cancer and perhaps other solid tumors.

## Supporting Information

Figure S1 **siRNA-mediated knockdown of DCLK1 results in downregulation of c-MYC in pancreatic tumor xenografts.** A and B, A decreased expression of c-MYC mRNA (using quantitative real-time RT-PCR) and protein (using Western blot) was observed in AsPC-1 tumor xenografts following knockdown of DCLK1. C, Decreased expression of c-MYC protein (brown) was observed following the knockdown of DCLK1 by immunohistochemical analyses. For bar graph in A, values are given as average ± SEM, and asterisks denote statistically significant differences (*p < 0.01) compared with Control (NP alone).
(TIF)

Figure S2 **NPsiRNA-mediated knockdown of DCLK1 downregulates NOTCH1 via *miR-144*.** A, siRNA-mediated knockdown of DCLK1 decreases NOTCH1 mRNA in AsPC-1 tumor xenografts. B, A decrease in NOTCH1 protein by immunohistochemical analysis was also observed. C, Knockdown of DCLK1 results in increased expression of *pri-miR-144* miRNA in tumor xenografts. For bar graph in A and C, values are given as average ± SEM, and *asterisks* denote statistically significant differences (*p < 0.01) compared with Control (NP alone).
(TIF)

Figure S3 **NPsiRNA-mediated knockdown of DCLK1 downregulates c-MYC via *Let-7a*, NOTCH1 via *miR-144* and pluripotency factors via *miR-143/145* in BxPC-3 cells.** siRNA-mediated knockdown of DCLK1 in BxPC-3 results in decreased expression of DCLK1 mRNA (A), increased expression *Let-7a, miR-144* and *miR-143/145* (B), decreased expression of c-MYC and NOTCH1 (C), and decreased expression of NANOG, KLF4, OCT4 and SOX2 (D).
(TIF)

Figure S4 **NPsiRNA-mediated knockdown of DCLK1 downregulates EMT transcription factors ZEB1, ZEB2 and angiogenic factors VEGFR1 and VEGFR2 via *miR-200* in BxPC-3 cells.** siRNA-mediated knockdown of DCLK1 in BxPC-3 cells results in decreased expression of *miR-200a, miR-200b* and *miR-200c* (A), decreased expression of ZEB1 and ZEB2 (B), and decreased expression of VEGFR1 and VEGFR2 (C).
(TIF)

Text S1 **Supporting methods.**
(DOCX)
