# Supplementary figures and images for: DCLK1 Regulates Pluripotency and Angiogenic Factors via microRNA-Dependent Mechanisms in Pancreatic Cancer
Source: PLoS One. 2013 Sep 9;8(9):e73940. doi: 10.1371/journal.pone.0073940 (PMC3767662; doi:10.1371/journal.pone.0073940)

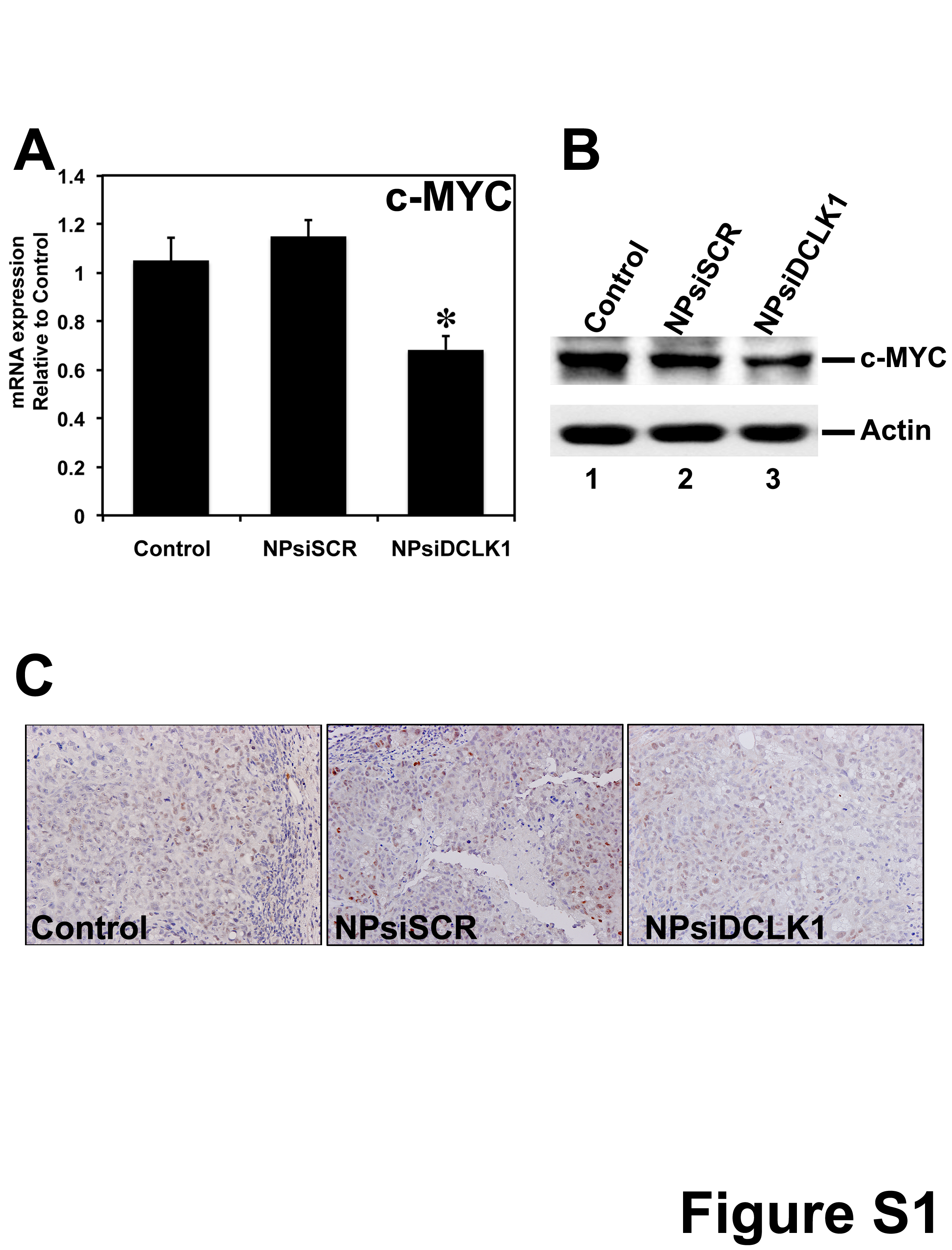

Supplement: Figure S1 — siRNA-mediated knockdown of DCLK1 results in downregulation of c-MYC in pancreatic tumor xenografts. A and B, A decreased expression of c-MYC mRNA (using quantitative real-time RT-PCR) and protein (using Western blot) was observed in AsPC-1 tumor xenografts following knockdown of DCLK1. C, Decreased expression of c-MYC protein (brown) was observed following the knockdown of DCLK1 by immunohistochemical analyses. For bar graph in A, values are given as average ± SEM, and asterisks denote statistically significant differences (*p < 0.01) compared with Control (NP alone). (TIF) [file pone.0073940.s001.tif]

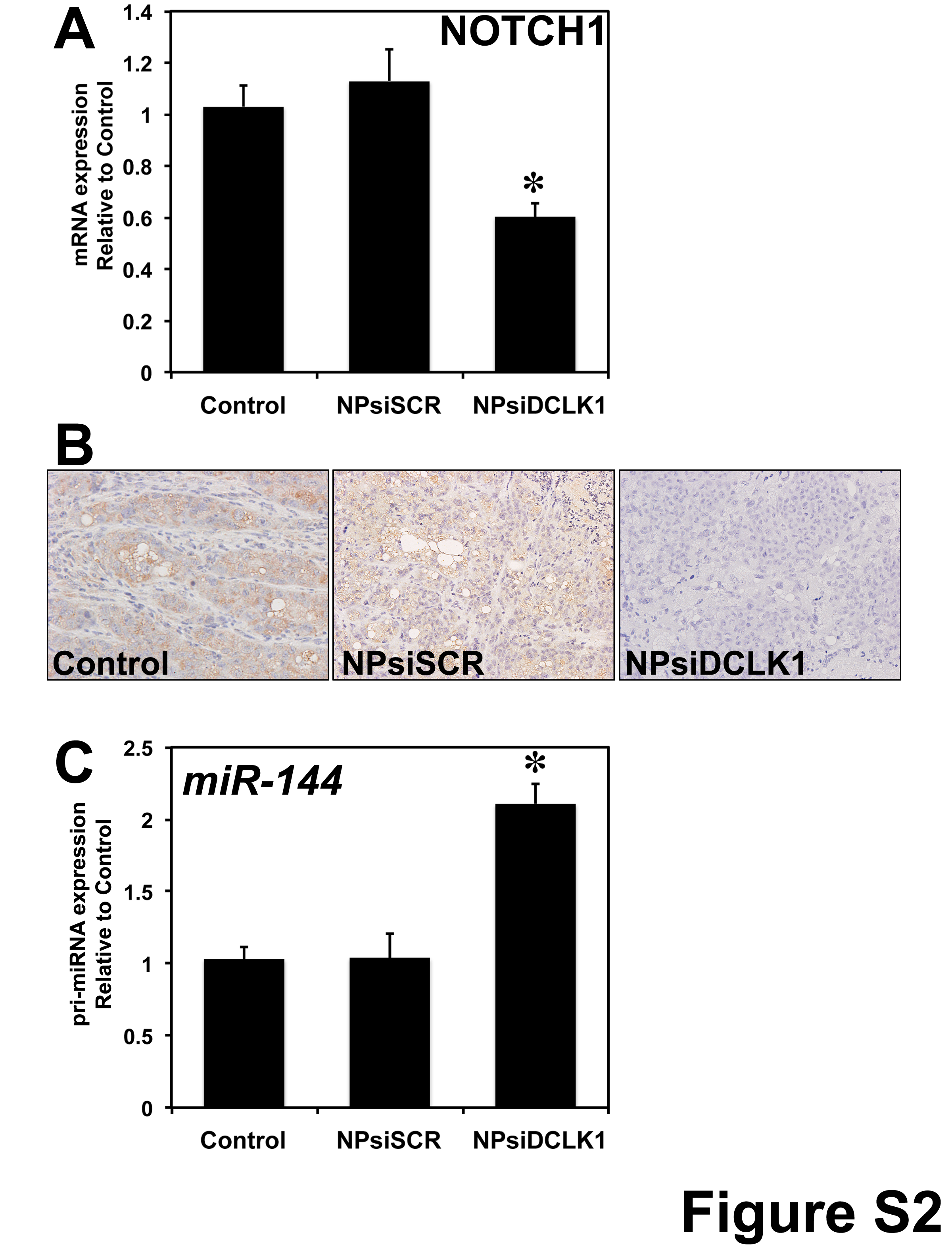

Supplement: Figure S2 — NPsiRNA-mediated knockdown of DCLK1 downregulates NOTCH1 via miR-144. A, siRNA-mediated knockdown of DCLK1 decreases NOTCH1 mRNA in AsPC-1 tumor xenografts. B, A decrease in NOTCH1 protein by immunohistochemical analysis was also observed. C, Knockdown of DCLK1 results in increased expression of pri-miR-144 miRNA in tumor xenografts. For bar graph in A and C, values are given as average ± SEM, and asterisks denote statistically significant differences (*p < 0.01) compared with Control (NP alone). (TIF) [file pone.0073940.s002.tif]

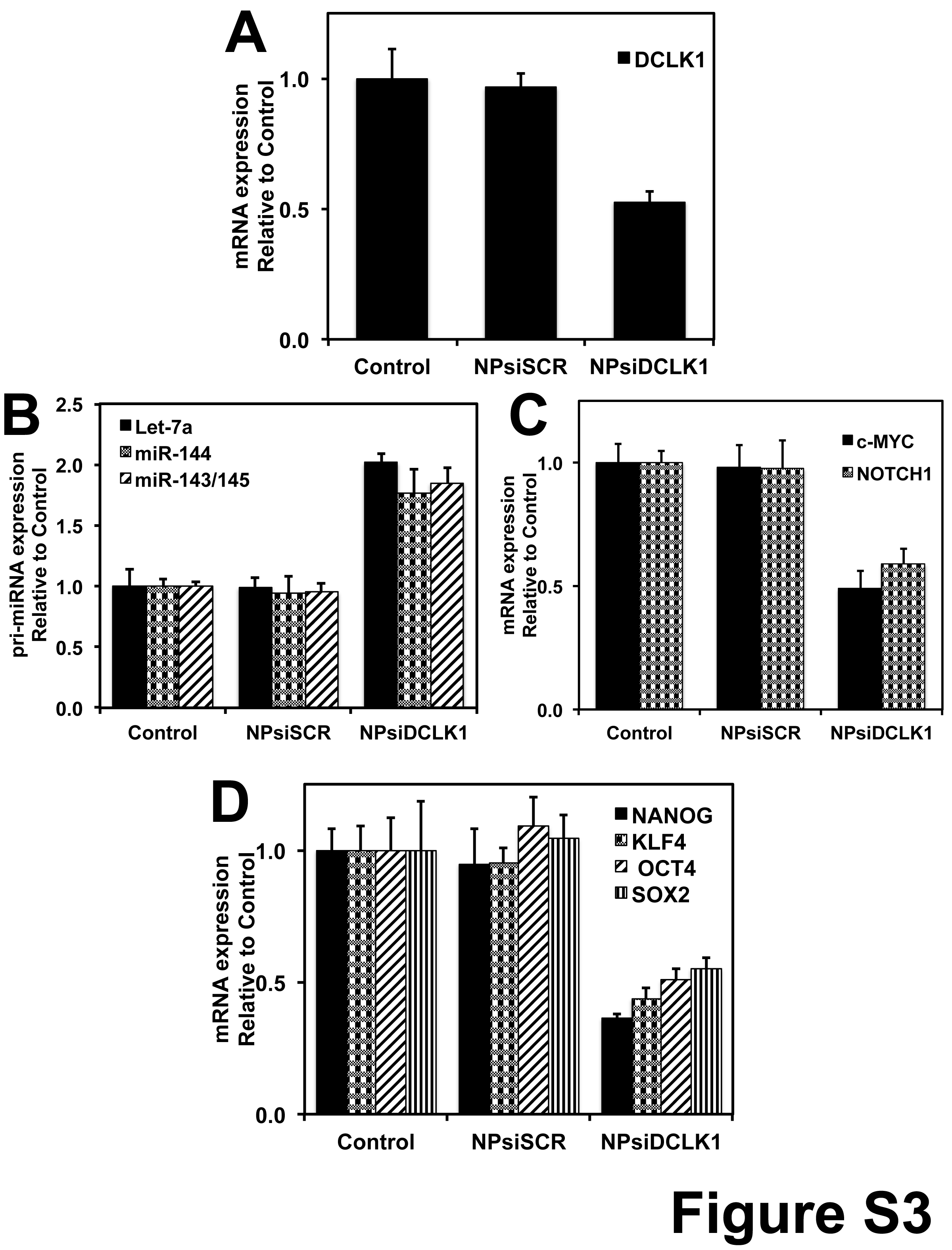

Supplement: Figure S3 — NPsiRNA-mediated knockdown of DCLK1 downregulates c-MYC via Let-7a, NOTCH1 via miR-144 and pluripotency factors via miR-143/145 in BxPC-3 cells. siRNA-mediated knockdown of DCLK1 in BxPC-3 results in decreased expression of DCLK1 mRNA (A), increased expression Let-7a, miR-144 and miR-143/145 (B), decreased expression of c-MYC and NOTCH1 (C), and decreased expression of NANOG, KLF4, OCT4 and SOX2 (D). (TIF) [file pone.0073940.s003.tif]

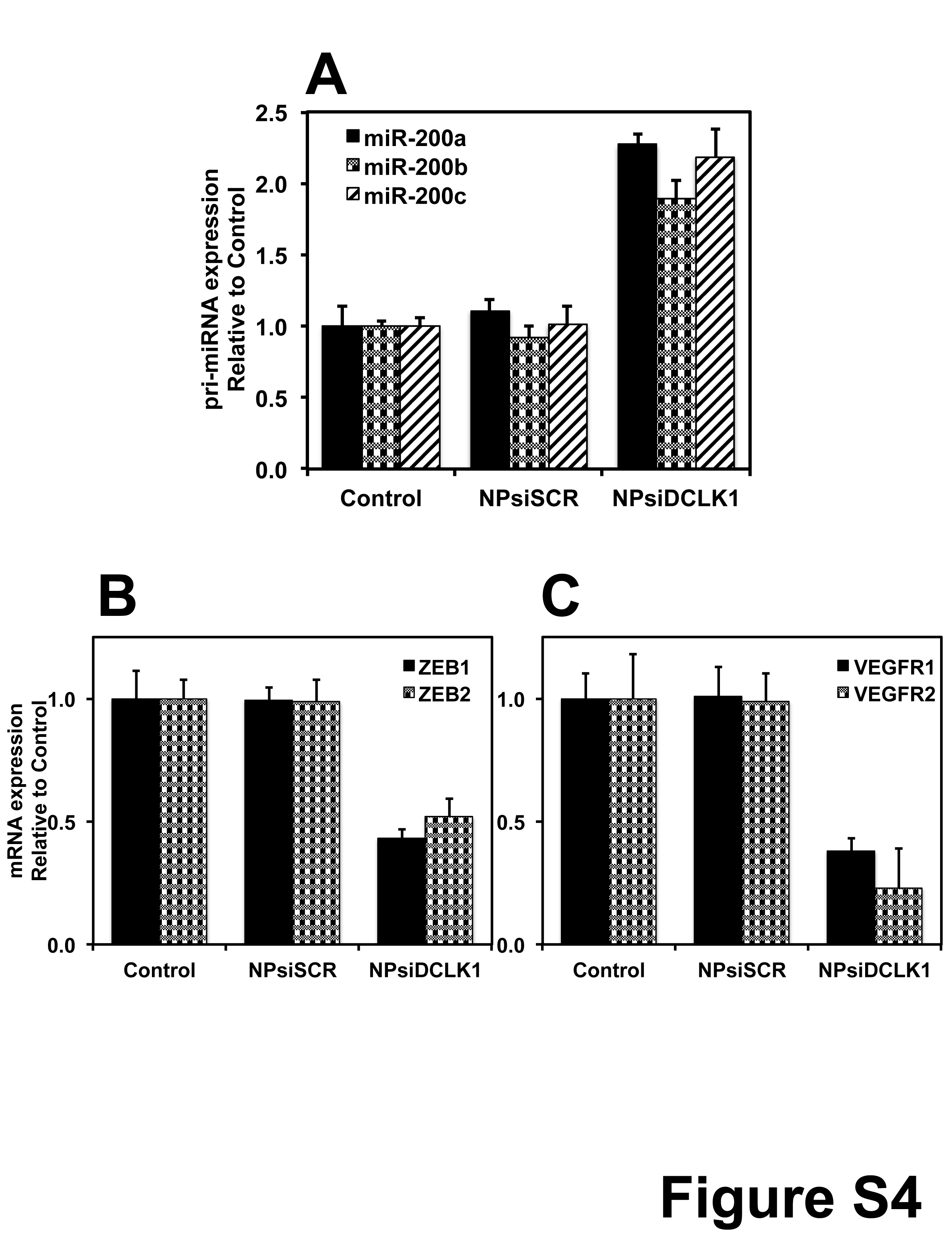

Supplement: Figure S4 — NPsiRNA-mediated knockdown of DCLK1 downregulates EMT transcription factors ZEB1, ZEB2 and angiogenic factors VEGFR1 and VEGFR2 via miR-200 in BxPC-3 cells. siRNA-mediated knockdown of DCLK1 in BxPC-3 cells results in decreased expression of miR-200a, miR-200b and miR-200c (A), decreased expression of ZEB1 and ZEB2 (B), and decreased expression of VEGFR1 and VEGFR2 (C). (TIF) [file pone.0073940.s004.tif]
