# Supplementary material for: DCLK1 Regulates Pluripotency and Angiogenic Factors via microRNA-Dependent Mechanisms in Pancreatic Cancer
Source: PLoS One. 2013 Sep 9;8(9):e73940. doi: 10.1371/journal.pone.0073940 (PMC3767662; doi:10.1371/journal.pone.0073940)
Supplement: Text S1 — Supporting methods. (DOCX) [file pone.0073940.s005.docx]

**Supplemental Methods – Text S1:**

**Reagents**

All cell culture reagents were purchased from Sigma Aldrich (St. Louis, MO, USA). PLGA was purchased from Lakeshore Biomaterials (Birmingham, AL, USA) as a 50:50 monomer ratio with a molecular weight of 58 kDa and inherent viscosity of 0.43 dl/g.

**Cell culture**

Human pancreatic cancer AsPC-1 and BxPC-3 cells were obtained from the American Type Culture Collection and propagated in Dulbecco’s modified Eagle medium supplemented with 10% fetal bovine serum and 1% penicillin-streptomycin in a humidified chamber at 37°C and 5% CO_2_.

**Immunohistochemical analysis**

Heat-induced epitope retrieval was performed on 4-μm formalin-fixed, paraffin-embedded sections utilizing a pressurized Decloaking Chamber (Biocare Medical LLC, Concord, CA) in citrate buffer (pH 6.0) at 99°C for 18 minutes. **Brightfield:** Slides were incubated in 3% hydrogen peroxide at room temperature for 10 minutes. After incubation with primary antibody [Nanog and KLF4 (Abcam Inc., Cambridge, MA), c-Myc (Santa Cruz Biotechnologies Inc., Santa Cruz, CA) or Notch-1 (Santa Cruz Biotechnologies)] overnight at 4°C, slides were incubated in Promark peroxidase-conjugated polymer detection system (Biocare Medical LLC) for 30min at room temperature. After washing, slides were devolved with Diaminobenzidine (Sigma–Aldrich). **Fluorescence:** Slides were incubated in a normal serum and BSA blocking step at room temperature for 20 minutes. After incubation with primary antibody [VEGFR1 and VEGFR2 (Santa Cruz Biotechnologies Inc., Santa Cruz, CA)] overnight at 4°C, slides were labeled with Alexa Fluor dye-conjugated secondary antibody and mounted with ProLong Gold (Invitrogen). **Microscopic Examination:** Slides were examined utilizing a Nikon 80i microscope and DXM1200C camera for brightfield analysis. Fluorescent images were taken with PlanFluoro objectives, utilizing CoolSnap ES2 camera (Photometrics). Images were captured utilizing NIS-Elements software (Nikon).

**Real-time Reverse Transcription-Polymerase Chain Reaction analyses**

Total RNA isolated from tumor xenografts and cancer cells was subjected to reverse transcription using Superscript™ II RNase H-Reverse Transcriptase and random hexanucleotide primers (Invitrogen, Carlsbad, CA). The complementary DNA (cDNA) was subsequently used to perform real-time polymerase chain reaction (PCR) by SYBR™ chemistry (SYBR Green I, Molecular Probes, Eugene, OR) for specific transcripts using gene-specific primers and JumpStart™ Taq DNA polymerase (Sigma-Aldrich). The crossing threshold value assessed by real-time PCR was noted for the transcripts and normalized with β-actin messenger RNA (mRNA). The quantitative changes in mRNA were expressed as fold-change relative to control with ± SEM value.

The following primers were used:

β-actin: forward: 5'-GGTGATCCACATCTGCTGGAA-3',

reverse: 5'-ATCATTGCTCCTCCTCAGGG-3';

DCLK1: forward: 5'- CAGCAACCAGGAATGTATTGGA -3',

reverse: 5'- ctcaactcggaatcggaagact-3';

c-Myc: forward: 5'-CACACATCAGCACAACTACGCA-3',

reverse: 5'-TTGACCCTCTTGGCAGCAG-3';

Notch-1: forward: 5'-CGGGTCCACCAGTTTGAATG-3',

reverse: 5'-GTTGTATTGGTTCGGCACCAT-3'.

KRAS: forward: 5'- -3',

reverse: 5'-GTTGTATTGGTTCGGCACCAT-3'.

ZEB1: forward: 5'-AAGAATTCACAGTGGAGAGAAGCCA-3',

reverse: 5'-CGTTTCTTGCAGTTTGGGCATT-3';

ZEB2: forward: 5'-AGCCGATCATGGCGGATGGC-3',

reverse: 5'-TTCCTCCTGCTGGGATTGGCTTG-3';

Snail: forward: 5'-AAGGCCTTCTCTAGGCCCT-3',

reverse: 5'-CGCAGGTTGGAGCGGTCAG-3';

Slug: forward: 5'-TGCTTCAAGGACACATTA-3',

reverse: 5'-CAGTGGTATTTCTTTAC-3';

Nanog: forward: 5'-ACCAGAACTGTGTTCTCTTCCACC-3',

reverse: 5'-CCATTGCTATTCTTCGGCCAGTTG-3';

KLF4: forward: 5'-CCAATTACCCATCCTTCCTG-3',

reverse: 5'-CGATCGTCTTCCCCTCTTTG-3';

OCT4: forward: 5'-AAGCGATCAAGCAGCGACTAT-3',

reverse: 5'-GGAAAGGGACCGAGGAGTACA-3';

SOX2: forward: 5'-CGAGATAAACATGGCAATCAAAAT-3',

reverse: 5'-AATTCGCAAGAAGCCTCTCCTT-3';

RREB1: forward: 5'-CTGGCGAGAGGCCTTACAAG-3',

reverse: 5'-CTACGTTTCAGAGGAGATGGA-3';

LIN28B: forward: 5’-GATGTATTTGTACACCAA-3’

reverse: 5’-TACCCGTATTGACTCAAGGCC-5’

**miRNA Analysis**

Total RNA isolated from tumor xenografts and cancer cells was subjected to reverse transcription with Superscript II RNase H-Reverse Transcriptase and random hexanucleotide primers (Invitrogen). The cDNA was subsequently used to perform real-time PCR by SYBR chemistry for *pri-let-7a, pri-miR-144, pri-miR-200a* and *pri-miR-145* transcripts using specific primers and JumpStart Taq DNA polymerase. The crossing threshold value assessed by real-time PCR was noted for *pri-let-7a, pri-miR-144,* and *pri-miR-200a* miRNAs and normalized with *U6* pri-miRNA. The changes in pri-miRNAs were expressed as fold-change relative to control with ± SEM values [^1^](#_ENREF_1).

The following primers were used:

*pri-U6*: forward: 5'-CTCGCTTCGGCAGCACA-3',

reverse: 5'-AACGCTTCACGAATTTGCGT-3';

*pri-let-7a*: forward: 5'-GAGGTAGTAGGTTGTATAGTTTAGAA-3',

reverse: 5'-AAAGCTAGGAGGCTGTACA-3';

*pri-miR-144*: forward: 5'-GCTGGGATATCATCATATACTG-3',

reverse: 5'-CGGACTAGTACATCATCTATACTG-3';

*pri-miR-200a*: forward: 5'-TTCCACAGCAGCCCCTG-3',

reverse: 5'-GATGTGCCTCGGTGGTGT-3'.

*pri-miR-143/145*: forward: 5'-AGGGCCAGCAGCAGGC-3',

reverse: 5'-TCAGGAAATGTCTCTGGCTGTG-3'.

*pri-miR-145*: forward: 5'-GGATGCAGAAGAGAACTCCA-3',

reverse: 5'-CCTCATCCTGTGAGCCAG-3'.

**Western blot analysis**

Tumor xenograft samples treated with siRNA-NPs were lysed and the concentration of protein was determined by the BCA protein assay kit (Pierce Biotechnology Inc., Rockford, IL). Forty μg of the protein was size separated in a 7.5-15% SDS polyacrylamide gel and transferred onto a nitrocellulose membrane with a semidry transfer apparatus (Amersham-Pharmacia, Piscataway, NJ). The membrane was blocked in 5% non-fat dry milk for 1 h and probed overnight with rabbit anti-c-Myc (Cell Signaling Danvers, MA) or rabbit anti-VEGFR1 (Santa Cruz Biotechnologies Inc., Santa Cruz, CA). Actin, used as a loading control was identified using a goat polyclonal IgG (Santa Cruz Biotechnology Inc). Subsequently, the membrane was incubated with anti-rabbit or anti-goat IgG horseradish peroxidase-conjugated antibodies (Amersham-Pharmacia) for 1 h at room temperature. The proteins were detected using ECL^TM^ Western Blotting detection reagents (Amersham-Pharmacia).

**Cell Invasion Assay**

AsPC-1 cells were treated with NPsiSCR or NPsiDCLK1 for 48h and subjected to invasion assay using the BD BioCoat^TM^ Tumor Invasion Assay System (BD Biosciences, Bedford, MA). 5000 cells were seeded with serum-free medium (containing NPsiRNA) into the upper chamber of the system. Bottom wells in the system were filled with growth media containing 10% FBS. After 24 h of incubation, the cells in the upper chamber were removed, and the cells that had invaded through Matrigel matrix membrane was fixed with methanol and stained with 1% toluidine blue and 1% borax for 2 min. This was destained with distilled water and mounted on slide and counted for invading cells (stained blue). 5 fields were counted on each insert (a total of 3 inserts per treatment) at 10X magnification.

**Luciferase reporter gene assay**

AsPC-1 cells were transfected with a plasmid containing the firefly luciferase (*Photinus pyralis*) gene with a complementary *miR-145* and *let-7a* (separate plasmids) binding site at its’ 3’ UTR obtained from Signosis Inc. (Sunnyvale, CA). The cells were also co-transfected with the *Renilla* luciferase expressing plasmid pRL-TK (Promega) as an internal control. Following transfection, the cells were treated with NPs alone, NP-siSCR, or NP-siDCLK1 and subjected to luciferase activity measurement. Luciferase activity was determined as per the manufacturer’s instructions (Dual-Luciferase Reporter Assay System; Promega) using a Biotek Synergy HT multi plate reader (BioTek, Winooski, VT) as described previously [^1^](#_ENREF_1)^,^ [^2^](#_ENREF_2).

Plasmids containing binding sites for *miR-200a, miR-200b, miR-200c* at the 3’UTR of firefly luciferase gene and plasmids with luciferase gene under the control of VEGFR1 and VEGFR2 3’UTR were obtained from Switchgear genomics (Menlo Park, CA). AsPC-1 cells were transfected with the above said plasmids along with pRL-TK. Following transfection, the cells were treated with NPs, NP-siSCR or NP-siDCLK1 and subjected to luciferase activity measurement (according to manufacturer’s instructions) using Bioteck Synergy HT multi plate reader.

The activity, normalized to *Renilla* luciferase activity, is presented as relative luciferase units relative to control with ± SEM values. Assays were performed in triplicate wells and experiments were repeated three times.

**Statistical analysis**

All experiments were performed in triplicates. Results are reported as average ± SEM unless otherwise indicated. Data were analyzed using the Student’s *t*-test. Results were considered statistically significant when *p* < 0.01.

**References:**

1. Sureban SM, May R, Ramalingam S, et al. Selective blockade of DCAMKL-1 results in tumor growth arrest by a *Let-7a* MicroRNA-dependent mechanism. Gastroenterology 2009;137:649-59, 659 e1-2.

2. Sureban SM, May R, Lightfoot SA, et al. DCAMKL-1 regulates epithelial-mesenchymal transition in human pancreatic cells through a *miR-200a*-dependent mechanism. Cancer Res 2011;71:2328-38.
